# Antiphotoaging effect of boiled abalone residual peptide ATPGDEG on UVB-induced keratinocyte HaCaT cells

**DOI:** 10.29219/fnr.v63.3508

**Published:** 2019-11-08

**Authors:** Jiali Chen, Peng Liang, Zhenbang Xiao, Mei-Fang Chen, Fang Gong, Chengyong Li, Chunxia Zhou, Pengzhi Hong, Won-Kyo Jung, Zhong-Ji Qian

**Affiliations:** 1 School of Chemistry and Environmental Science, Guangdong Ocean University, Zhanjiang, P. R. China; 2 College of Food Science and Technology, Guangdong Ocean University, Zhanjiang, P. R. China; 3 Shenzhen Institute, Guangdong Ocean University, Shenzhen, P. R. China; 4 Department of Biomedical Engineering, and Center for Marine Integrated Biomedical Technology (BK21 Plus), Pukyong National University, Busan, Republic of Korea

**Keywords:** abalone by-products, molecular docking, MAPKs, photoaging, type I procollagen, HaCaT cells

## Abstract

**Introduction:**

A previous study has shown that Ala–Thr–Pro–Gly–Asp–Glu–Gly (ATPGDEG) peptide identified from boiled abalone by-products has high antioxidant activities and antihypertensive effect.

**Objective:**

In this study, we further investigated its antiphotoaging activities by ultraviolet B (UVB)-induced HaCaT cells.

**Result:**

UVB irradiation significantly increased the content of intercellular reactive oxygen species (ROS) and the production of matrix metalloproteinases (MMPs) in HaCaT cells and decreased its content of collagen. First, the generation of intercellular ROS was reduced by abalone peptide in UVB-induced HaCaT cells. And activities of MMP-1 and MMP-9 were reduced by abalone peptide in a dose-dependent manner. Furthermore, western blot analysis demonstrated that abalone peptide downregulated the expression of p38, c-Jun N-terminal kinases, and extracellular signal-regulated kinases via mitogen-activated protein kinases (MAPKs) and NF-κB signaling to protect type I pro collagen and DNA damage. Molecular docking simulation confirms that abalone peptide inhibited activities of MMP-1 and MMP-9 by docking their active site, among them N-terminal Ala, C-terminal Gly, and Pro at the third position of N-terminal made a great contribution.

**Conclusion and recommendation:**

Abalone peptide could protect type I procollagen synthesis in UVB-irradiated HaCaT cells, and it is a potential peptide for the treatment of skin photoaging in the future.

## Popular scientific summary

- Abalone is a marine gastropod with high nutritional value, this mainly attributed to the bioactive material present in its composition, and is considered as a valuable resource with potential applications worldwide.
- This study demonstrates that abalone peptide from boiled abalone by-products inhibits ROS production, MMP-1 and MMP-9 secretion; suppresses MAPKs and NF-κB pathways phosphorylations; and protects type I procollagen I synthesis in UVB-irradiated HaCaT cells.
- Abalone peptide is a potential bioactive peptide for the treatment of skin photoaging in the future.

One of the most prominent characteristics of skin aging is the destruction of skin integrity occurred by exposure to environment. Skin aging can be classified into intrinsic aging and extrinsic aging. The intrinsic aging (also called chronologic aging) is the process of senescence that affects all body organs, the extrinsic aging, also called photoaging (1), is an aging procedure happened by exposure of skin to ultraviolet radiation (UVR) (2). As one of the most extrinsic aging factors, solar UV includes that long wavelength ultraviolet A (UVA, 320–400 nm), middle wavelength ultraviolet B (UVB, 280–320 nm), and short wavelength ultraviolet C (UVC, 100–280 nm) (3). Particularly, UVB rays are the most damaging and genotoxic to humans, while UVA rays represent a relatively low-energy source of radiation, and UVC rays are generally absorbed by the ozone layer and thus cannot reach human skin (4).

Primarily, photoaged skin tissue are altered dermal connective, it may give rise to alterations of extracellular matrix (ECM) and loss of skin structure due to disorganized and damaged collagen fibrils and reduction of procollagen synthesis (5). Skin exposure to UVB radiation gives rise to alterations in the ECM, which further expedites skin aging for the augment of matrix metalloproteinases (MMPs) (6); the expression of MMPs is one of the important indicators of photoaging of skin. MMPs as a family of zinc-dependent proteolytic enzymes (7) include MMP-1, MMP-3, and MMP-9, have the ability to degrade collagen and other ECM proteins, and play a crucial role in different pathophysiology conditions, such as invasion, vascular remodeling, angiogenesis, and tumor metastasis (8, 9). However, the overexpression of MMPs results in a wide range of physiological and pathological processes (10); the degraded collagen fragments generated from MMPs would downregulate the synthesis of new collagens (11). In particular, MMP-1 is the most vital MMP in the degradation of the ECM by photoaging, it is mainly responsible for the degradation of the most abundant collagen type I which supported structure in the dermis (12–15). Besides being responsible for normal turnover of the ECM in connective tissue (16), it is further responsible for the keratinocyte migration during wound repair (17). After MMP-1, MMP-9 further decomposes collagen fragments (18). MMP-9 degrades the ECM and influences skin wrinkle formation and skin thickness (19). A growing body of research have identified that loss of structural integrity of ECM is considered to be the main cause of pathogenesis of skin photoaging, its main structural component is type I procollagen, and MMP-1 specifically degrades type I procollagen during photoaging; therefore, the generation of MMPs and debasement of type I procollagen in photoaging play profound roles (20, 21). The expression of MMPs is as important as the regulation of transcription factors and pathways. Studies have found that oxidative stress induced by UV can further mediate the phosphorylation of its upstream signaling protein kinases, for example, mitogen-activated protein kinases (22, 23). In signaling pathways for the regulation of activation of NF-κB and MMP-1 expression, mitogen-activated protein kinases (MAPKs) signal transduction pathways including that incorporate extracellular signal-regulated kinases (ERK), c-Jun N-terminal kinases (JNK), and p38, all play fundamental roles (24, 25). They are necessary for transcriptional regulation of MMP-1, MMP-3, and MMP-9, resulting in collagen degradation. Among them ERK is beneficial to stimulate the expression of c-Fos, whereas p38 and JNK activation are important for the expression of c-Jun. c-Jun in combination with c-Fos forms the transcription factor AP-1, which is the key of regulation (26–28).

After UVB irradiation activates phosphorylation of ERK, JNK, and p38 kinases, these activated MAPKs also affect the activation of p65 subunit which is the critical component of the NF-κB transcription factor (29). Besides, DNA damage caused by UV radiation directly and indirectly results in skin cancer and photoaging (30).

It is extremely important to suppress photoaging in the skin to reduce the expression and activity of MMP-1 and MMP-9. Many studies have shown that the screening key for numerous therapeutic agents mainly depends on the ability to inhibit production of MMP-1 and control of collagen metabolism. Nowadays, natural compounds with inhibitory ability of MMPs, especially antioxidants, have been successfully used to prevent photoaging (26). Hence, finding those MMP-1 and MMP-9 inhibitors with ROS scavenging activity seems a perspective treatment to increase the procollagen type I production for skin photoaging therapies.

In several studies, epidermal keratinocytes and dermal fibroblasts are major components of MMPs induced by UVR (31, 32). In human skin, keratinocytes act as a physical barrier to the external environment and protect against harmful external stimuli (33). The human keratinocytes (HaCaT) cells is a line of human keratinocytes, a study found that reported mRNA and protein of MMP are mainly originated from epidermis, specifically from keratinocytes, as stated by Fisher et al. (34).That is the reason why we choose HaCaT cells as UVB irradiation model in this study.

**Fig. 1:**
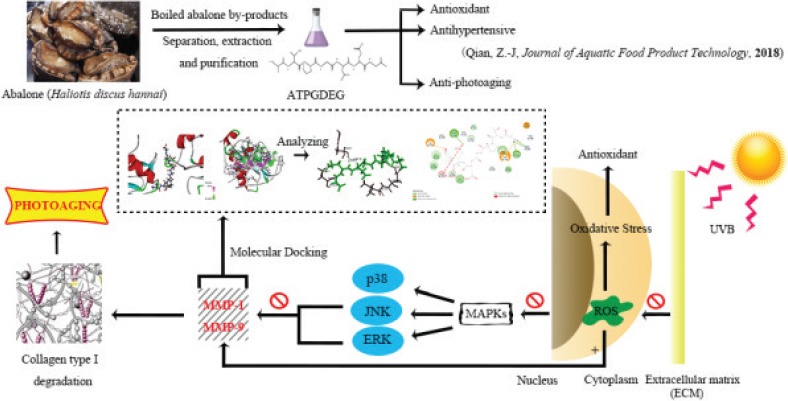
This research is about the protective effect of boiled abalone residual peptides against photoaging and it is a series of specific experiment design of activity identification. This figure summarized the separation and purification of the peptide and researched bioactivity in earlier study, further designed experiments to study the main pathways and matrix metalloproteinases (MMPs) involved in antiphotoaging.

In the last several years, attention has been turned to natural marine products whose new compounds contain antiphotoaging activities. Abalone (*Haliotis discus hannai*), as a marine gastropod with commercially high value and applications worldwide, has presented a steady growth tendency due to its production and consumption, and the global production of farmed abalone was estimated at 129, 287 metric tons (MT) in 2015 (35). Korea’s total yield of farmed abalone was estimated at 7, 580 MT in 2009; abalone farms in China raised their annual production from 42, 373 MT in 2010 to 115, 397 MT in 2015 (36). Multifarious products of abalone have been invented by boiling and steaming of abalone in the manufacturing process. Numerous manufacturing plants, however, discard the material left after abalone boiling process in most cases. And 20% salts and considerable concentrations of protein, carbohydrate, lipid, and minerals indwell in these boiled abalone by-products (BABs). Recently, BABs have been brought into research focus, its relevant methodological aspects and practical use are still under research. Some researchers have identified that antioxidant activity and ACE inhibitory activity, respectively, consist in abalone protein and abalone hydrolysate (37). Based on previous research, abalone peptide purified from BABs (38) had been identified for its antioxidant and antihypertensive effect, and our study is the first to demonstrate the antiphotoaging effect of abalone peptide by regulation of oxidative stress and matrix metalloproteinase activity. Fig. 1 portrays the relative research background and circumstance of abalone peptide from BABs and its approach and mechanism of anti-photoaging effect in detail.

## Materials and methods

### Reagents

The low-molecular-weight peptides from BABs (abalone peptide ATPGDEG, MW 752 Da) were obtained according to our previous experiment (38). Dulbecco’s modified Eagle’s medium (DMEM), fetal bovine serum (FBS), phosphate buffered saline (PBS), and trypsin (0.25%) were purchased from Gibco (Carlsbad, NY, USA).3-(4,5-Dimethylthiazol-2-yl)-2,5-diphenyltetrazolium bromide (MTT), dimethyl sulfoxide (DMSO), and 2’,7’-dichlorofluoresin diacetate (DCFH-DA) were purchased from Sigma Chemical Co. Ltd. (St. Louis, MO, USA). The Enzyme-linked immunosorbent assay (ELISA) kits of MMP-1, MMP-9, and procollagen I were purchased from R&D (Systems Inc., Minneapolis, MN, USA). The BCA protein assay kit was purchased from ThermoFisher Scientific Co Ltd. (Shanghai, China). Primary antibodies for β-actin, p-38, p38, p-JNK, JNK, p-ERK, ERK, and secondary antibodies were purchased from Santa Cruz Biotechnology (Santa Cruz, CA, USA). Secondary antibody conjugated to horseradish peroxidase (goat anti-rabbit IgG) was purchased from Abbkine (Redlands, CA, USA). Chemicals not mentioned in the present research were of analytic grade.

### Cell culture

The human keratinocyte cell lines (HaCaT cells) were purchased from Shanghai Fudan IBS Cell Resource Center and cultured in 5% CO_2_ at 37°C in DMEM with FBS (10%, v/v), penicillin (100 U/mL), and streptomycin (100 mg/L).

### Cell viability assay and UVB irradiation

HaCaT (1 × 10^4^ cells/well) cells were plated in 96-well plates containing DMEM with FBS (10%, v/v) and incubated in 5% CO_2_ at 37°C for 24 h. Cell viability assay was prepared according to MTT method (39). UVB irradiation was prepared as per the process of Choi et al. (40) with slight modifications. HaCaT cells were incubated in culture medium with abalone peptide (10, 20, 50, and 100 μM) for 24 h, then covered in a thin layer of PBS after removal of culture medium, and were irradiated with UVB at 30 mJ/cm^2^ (wavelength range: 300–320 nm; peak: 311 nm; with a UVB lamp [PL-S 9W/01; Royal Dutch Philips Electronics Ltd., Amsterdam, North Holland, Holland]). Irradiance was measured by UV illumination (UV-340A; Luchang Electronic Enterprise, Taipei, Taiwan). To remove PBS later, HaCaT cells were further incubated in culture medium with abalone peptide (10, 20, 50, and 100 μM) for 24 h. Cell viability was measured using MTT method as described previously.

### Determination of intracellular formation of ROS using DCFH-DA labeling

Intracellular formation of ROS was assessed according to a method described previously (41). HaCaT cells were grown in black microtiter 96-well plates and then treated with different concentrations of abalone peptide and incubated for 24 h; the UVB radiation was same as before. Then HaCaT cells were labeled with 10 μM DCFH-DA in serum-free medium and kept for 20 min in the dark. The formation of fluorescent dichlorofluorescein (DCF) due to oxidation of DCFH in the presence of ROS, it can be photoed at the excitation wavelength of 485 nm and the emission wavelength of 528 nm (GENios microplate reader, Tecan Austria GmbH, Grodig/Salzburg, Austria).

### ELISA of MMP-1, MMP-9, and type I procollagen

HaCaT cells were seeded at 2 × 10^4^ cells/well in six-well plates, and the UVB radiation was same as before. The culture supernatants were collected for the secretion of MMP-1, MMP-9, and type I procollagen by ELISA, which were performed according to the manufacturer’s protocol of the respective kits.

### Western blot of MAPKs and NF-κB pathways

The protein expression levels of MAPKs and NF-κB pathways were determined by western blotting analysis. HaCaT cells were seeded at 2 × 10^4^ cells/well in six-well plates, and the UVB radiation was same as before. First, the extracted protein was diluted by 10 times and mixed with the standard substance in proportion to the working fluid method. The absorbance was added into the standard curve to obtain the protein concentration. Cells were lysed in radio immunoprecipitation assay (RIPA) buffer (150 mM sodium chloride, 1% Triton X-100, 1% sodium deoxycholate, 0.1% sodium dodecyl sulfate [SDS], 50 mM Tris-HCl pH 7.5, and methylenediaminetetraacetic acid [EDTA]) containing proteinase inhibitor cocktails (Roche, Indianapolis, IN, USA). Protein concentrations were quantified with the rapid gold BCA protein assay kit (ThermoFisher Scientific Co Ltd., Shanghai, China). Proteins (30 μg/lane) were resolved with 8–12% SDS-polyacrylamide gel electrophoresis, and Western blot analysis was performed as described previously (42).

### Immunocytochemistry

The HaCaT cells were seeded in 24-well plates (5 × 10^4^ cells/well) in advance, treated as described above, and were then harvested. After washing thrice with PBS buffer, the cells were fixed in phosphate buffer solution containing 4% paraformaldehyde (4°C, 20 min). Then, permeabilization was carried out by using 0.2% Triton X-100 in PBS, followed by incubation (4°C, 10 min). Cells were then blocked with 5% bovine serum albumin (BSA) in PBS, removed, and directly incubated overnight at 4°C with anti-p65 antibody (1:100). After removing the primary antibody, the cells were washed again and incubated, in the dark and at room temperature for 3 h, with the corresponding goat anti-rabbit IgG secondary antibody (1:500; Abbkine, CA, USA). Finally, the nuclei were stained using DAPI (100 ng/mL) for 5 min. The images were then observed under an inverted fluorescence microscope (Olympus, Tokyo, Japan).

### Comet assay

Comet assay was performed as described previously (43). Briefly, cells were added with 0.5% low-melting point agarose, and then were put on a slide precoated with 1% normal agarose. After solidification, normal agarose was added to form an additional layer. Then, the cells were lysed in a prechilled detergent solution (1% Triton X-100, 2.5 M NaCl, 0.1 mM Na_2_EDTA, 0.1% sodium sarcosine, and 10 mM Tris, pH 10) for 1 h. The slides were placed in an electrophoresis chamber with an alkaline buffer (300 mM NaOH and 1 mM Na_2_EDTA) for unwinding and expression of alkali-labile sites (30 min) and subsequently electrophoresed (at 25 V and 300 mA for 20 min). The electrophoresis slides were stained with 15 μg/mL ethidium bromide for 15 min. Then comets were detected with a fluorescent microscope. A total of 100 cells were randomly selected for quantifying the DNA damage (% tail DNA/head DNA) using CASP software to analyze the comet images.

### Molecular docking

The three-dimensional structures of MMP-1 (PDB ID: 966C) and MMP-9 (PDB ID: 5CUH) were obtained from the Protein Data Bank. PDB ID: 5CUH was chosen because it had similar crystal structure as PDB ID: 1GKC, which is the first reported structure for MMP-9 bound to a reverse hydroxamate inhibitor. We used USES soft-core particles and optional grid representation to dock the ligand molecule (abalone peptide) with the active site of the diverse receptor (MMP-1 and MMP-9) upon the Discovery Studio 3.5 module CDOCKER charm-based docking program. First, the high-temperature dynamics method was used to randomly search for the small molecule conformation and then each conformation was optimized in the region of the active site of the receptor via simulated annealing method so as to make the docking results more accurate.

### Statistical analysis

All analyses were carried out on triplicate samples. GraphPad Prism 5 (GraphPad Software, San Diego, CA, USA) was used for the statistical analysis. Multiple-group comparisons were performed by the one-way ANOVA with *post hoc* testing. The differences were considered statistically significant at *P* < 0.05.

## Results

### Effect of abalone peptide on cell viability of UVB-induced HaCaT cells

**Fig. 2:**
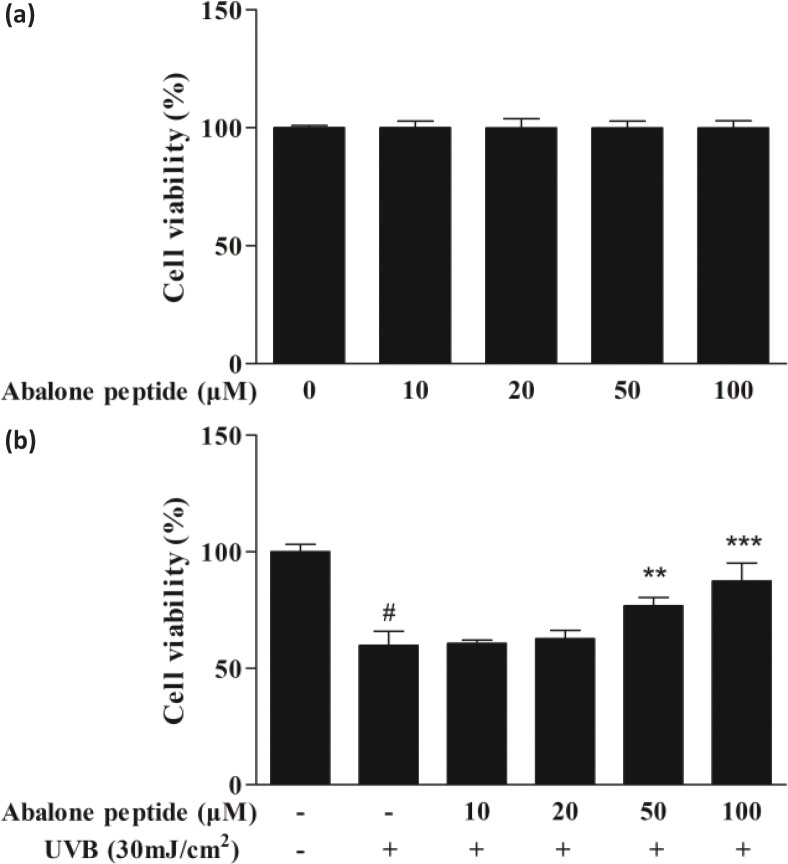
Effects of abalone peptide on the viability of HaCaT cells by MTT assay. (a) Effect of different concentrations (10, 20, 50, and 100 μM) of abalone peptide on cell viability of HaCaT cells. (b) Cell viability of ultraviolet B (UVB)-induced HaCaT cells at different concentrations (10, 20, 50, and 100 μM) of abalone peptide. Significance of the difference from blank at #*P* < 0.001; significance of the difference from control at **P < 0.01 and ***P < 0.001.

We evaluated the effect of abalone peptide on HaCaT cells after 24 h treatment and the cell viability was determined using MTT assay. Abalone peptide in the range of 10–100 μM is nontoxic (Fig. 2a); their cell viability is around 96.75–103.12%. As shown in Fig. 2b, the cell viability of HaCaT cells after exposure to UVB irradiation for 24 h significantly (*P* < 0.001) reduced to 59.79% compared with the UVB (−) group, significantly lower than the blank group (100%). Thus, the abalone peptide (50–100 μM) could efficiently enhance the cell viability of HaCaT cells damaged by UVB at various concentrations to 87.64% (*P* < 0.01, *P* < 0.001). The results indicated that abalone peptide had the protective effect for HaCaT cells by UVB radiation.

### Effect of abalone peptide on formation of ROS in UVB-induced HaCaT cells

**Fig. 3:**
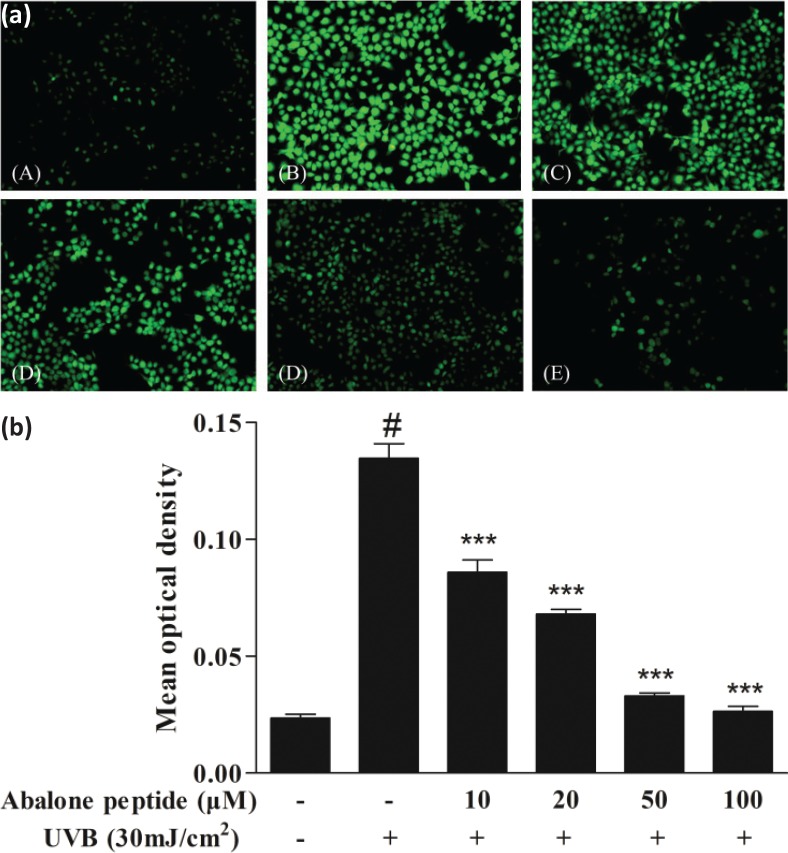
Effect of abalone peptide on the expression level of ROS fluorescence by DCFH-DA. (a) Representative image of ROS fluorescence staining of HaCaT cells with different treatment: (a) cells without treatment (the blank group); (b) cells exposed to 30 mJ/cm^2^ of UVB (the control group); (c–f) cells treated with abalone peptide (10, 20, 50, and 100 μM, respectively) and 30 mJ/cm^2^ of UVB. (b) Quantity of production of intracellular ROS by Image J. Significance of the difference from blank at #*P* < 0.001; significance of the difference from control at ***P < 0.001.

The intracellular ROS concentration was determined by measuring the intensity of DCFH fluorescence. After the cells labeled with DCFH-DA probe were cultured for 2 h, the intensity of fluorescence in the images indicates the amount of ROS produced in the cells (Fig. 3a). As shown in Fig. 3b, the mean optical density of DCFH fluorescence in HaCaT cells with UVB irradiation apparently increased to 0.13 ± 0.01 compared with the blank group (*P* < 0.001), whereas preincubation with abalone peptide (10–100 μM) significantly reduced the increased fluorescence to below 0.02 compared with the blank group (*P* < 0.001), suggesting that the addition of abalone peptide had a reducing effect on ROS generation.

### Effect of abalone peptide on secretion of MMP-1, MMP-9, and type I procollagen in UVB-induced HaCaT cells

**Fig. 4:**
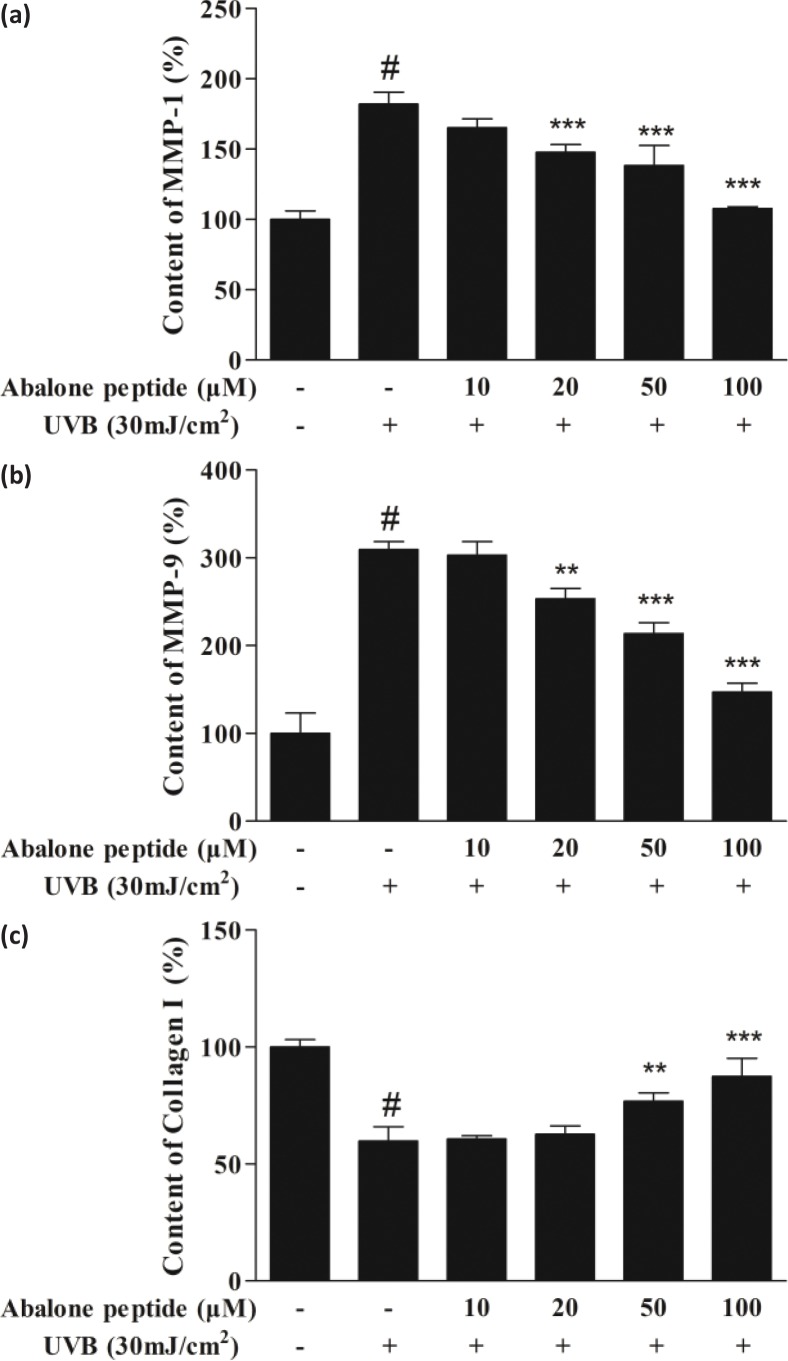
The effect of abalone peptide on the secretion levels of intercellular matrix metalloprpteinases (MMP)-1 (a), MMP-9 (b), and type I procollagen (c) in UVB-induced HaCaT cells by ELISA. Significance of the difference from blank at #*P* < 0.001, significance of the difference from control at **P < 0.01 and ***P < 0.001.

MMP-1 and MMP-9 play an important role in type I collagen synthesis, inhibition of its expression is a way to prevent or mitigate UV-induced skin aging, and secretion of type I procollagen is important to provide structural support of skin. As shown in Fig. 4a–b, UVB irradiation increased the secretion of MMP-1 and MMP-9 significantly (*P* < 0.001, from 54.92 ± 3.13% to 99.99 ± 4.07%, from 32.30 ± 8.42% to 99.99 ± 3.04%), while 50 μM of abalone peptide effectively reduced their secretion in UVB-induced HaCaT cells (*P* < 0.001). Secretion of type I procollagen was decreased from 167.25 ± 5.44% to 99.99 ± 9.76% (*P* < 0.001) after induction with 30 mJ/cm^2^ UVB. In contrast, 50 μM of abalone peptide effectively increased this reduction to 146.26 ± 10.29% (*P* < 0.001; Fig. 4c). Therefore, ELISA results can prove that more than 50 μM concentration of abalone peptide can increase the secretion of type I procollagen in cells, reduce the secretion of MMP-1 and MMP-9, and prevent skin aging.

### Effect of abalone peptide on phosphorylation level of MAPKs signaling pathway in UVB-induced HaCaT cells

**Fig. 5:**
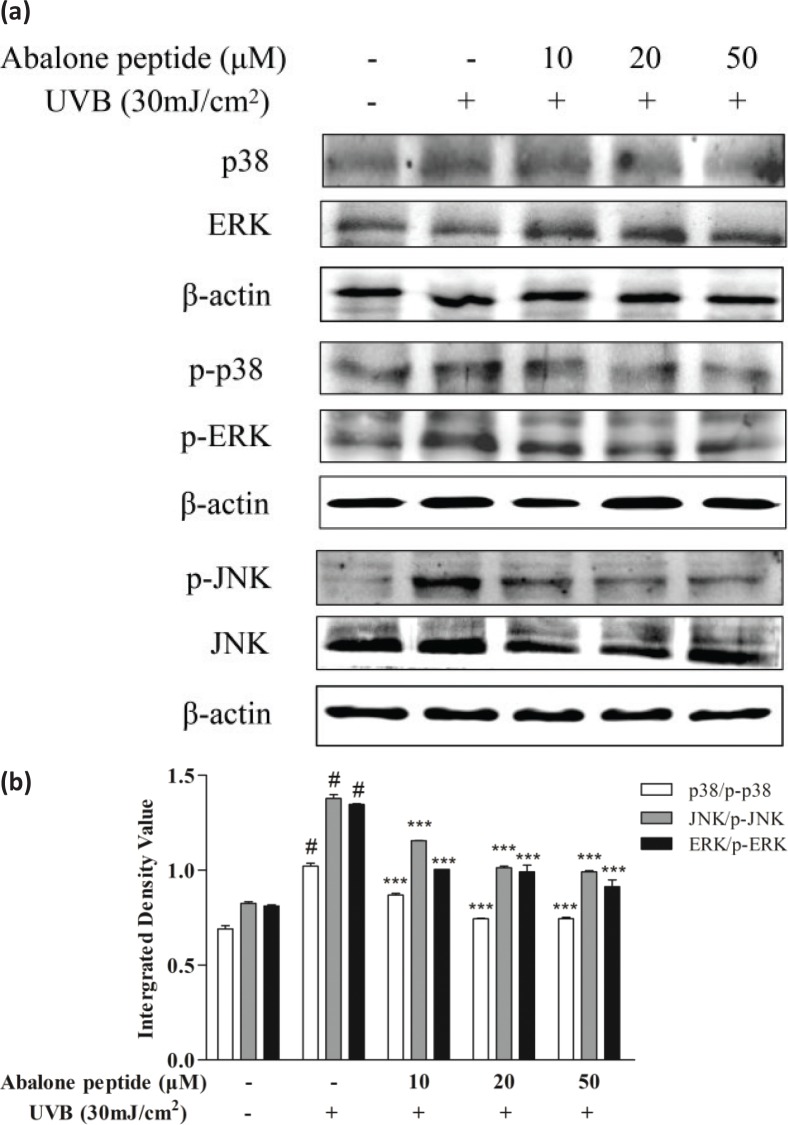
Abalone peptide alleviates the phosphorylation of mitogen-activated protein kinases (MAPKs) signaling pathway in UVB-induced HaCaT cells by Western blot analysis. (a) Western blot image of ERK, JNK, and p38 MAP kinases in UVB-induced HaCaT cells following different treatments; (b different group quantitative results of p38, JNK, and ERK. β-Actin was used as an internal control. Significance of the difference from blank at #*P* < 0.001; significance of the difference from control at ***P < 0.001.

As MAPKs signaling pathway was implicated in the transcriptional regulation of UVB-induced MMP-1 and MMP-9 expression, its phosphorylation expression level in UVB-irradiated HaCaT cells was measured (Fig. 5a). Western blot results showed that 30 mJ/cm^2^ UVB irradiation treatment in HaCaT cells induced the rapid phosphorylation of JNK, p38 MAPK, and ERK (Fig. 5b). The inhibitory effects of abalone peptide, thus, largely through MAPKs signaling pathway. However, this promoted phosphorylation of p38, JNK, and ERK was significantly attenuated by treatment with 10 μM abalone peptide (*P* < 0.001). Therefore, the results indicate that abalone peptide treatment attenuates MAPKs activation in the UVB-induced HaCaT cells.

### Effects of abalone peptide on the activation and nuclear translocation of NF-κB in UVB-stimulated HaCaT cells

**Fig. 6:**
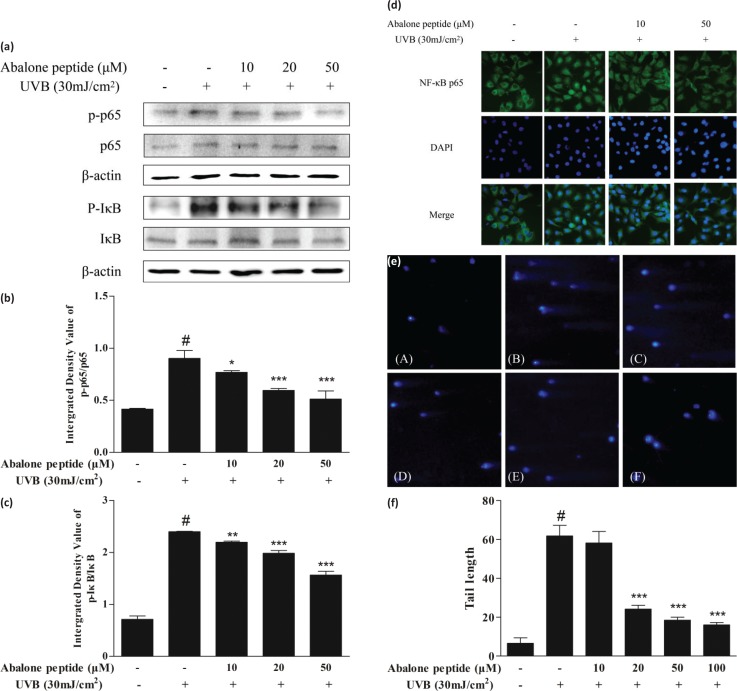
Abalone peptide alleviates the activation and nuclear translocation of NF-κB signaling pathway and prevents DNA damage in UVB-induced HaCaT cells by western blot, immunocytochemistry analysis, and comet assay. (a) Western blot image of NF-κB p65 and IκB in UVB-induced HaCaT cells following different treatments. Parts (b) and (c), respectively, represent the different group quantitative result. β-Actin was used as an internal control. (d) Fluorescent images of NF-κB p65 in UVB-induced HaCaT cells following different treatments. (e) Representative image of DAPI staining of HaCaT cells with different treatments: (A) cells without treatment (the blank group); (B) cells exposed to 30 mJ/cm^2^ of UVB (the control group); (C–F) cells treated with abalone peptide (10, 20, 50, and 100 μM, respectively) and 30 mJ/cm^2^ of UVB. (f) Quantitative results of different group in images. Significance of the difference from blank at #*P* < 0.001; significance of the difference from control at *P < 0.05; **P < 0.01, and ***P < 0.001.

To elucidate the mechanisms through which abalone peptide affects the expression of MMPs, the effects of abalone peptide on the activation of NF-κB were examined by western blot analysis and immunocytochemistry. The majority of the inhibitors of NF-κB activation exert their effects through the suppression of IκBα phosphorylation and degradation. In this study, we found that abalone peptide inhibited the UVB-induced phosphorylation and degradation of NF-κB p65 and IκBα, as well as the nuclear translocation of p65 NF-κB (Fig. 6a–d).

### Abalone peptide prevents UVB-induced DNA damage in HaCaT cells

Protective effects of abalone peptide against UVB-induced DNA damage were measured by the comet assay. As shown in Fig. 6e, clear comets were observed in UVB radiation treatment compared with normal cells; comet tail formation was apparently increased after exposure of the cells to UVB radiation, represented by broken DNA fragments migrating out of the nucleus. As represented in other fluorescence images, however, abalone peptide pretreatment followed by UVB exposure significantly reduced the comet tail. Comparing with UV-irradiated group, abalone peptide pretreatment significantly reduced UVB-induced DNA damage (*P* < 0.001; Fig. 6f).

### Molecular docking analysis

**Fig. 7:**
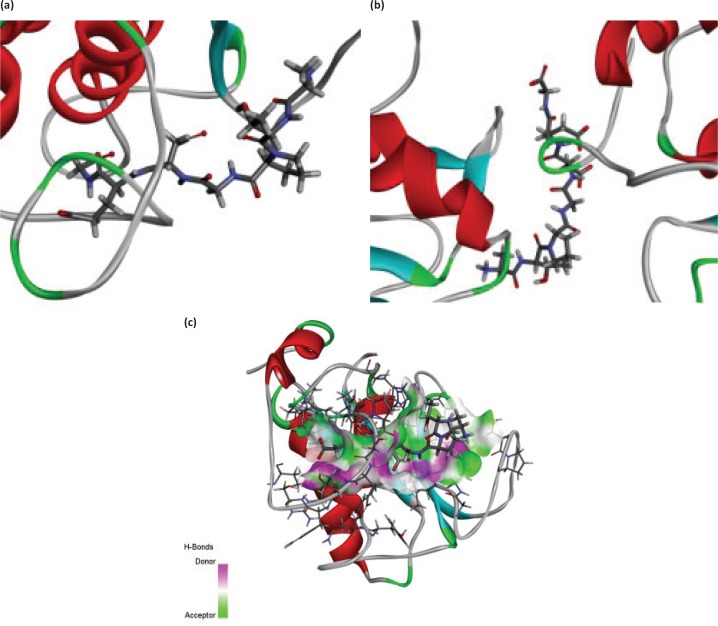

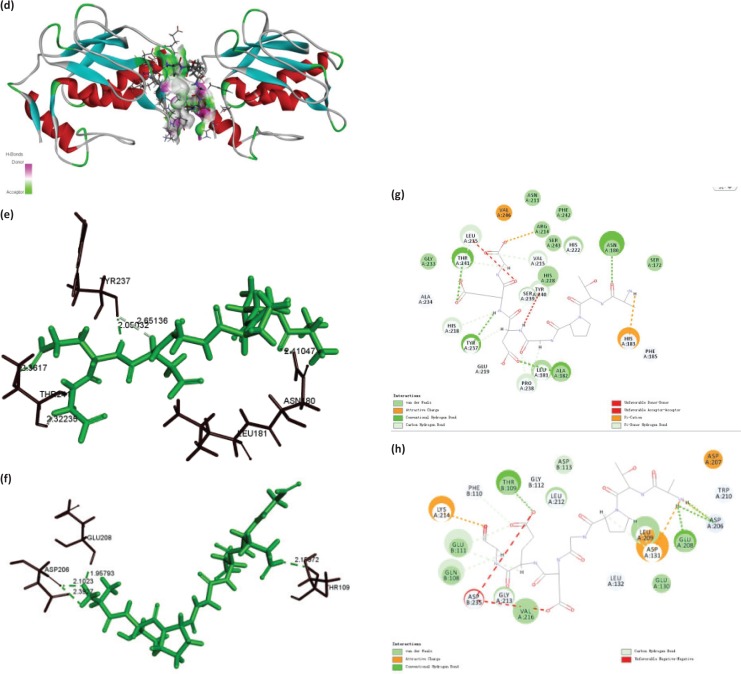
The molecular docking of abalone peptide with MMP-1 and MMP-9, respectively. Local overview poses of the best interaction after automated docking of ATPGDEG-MMPs: (a) MMP-1, (b) MMP-9. Parts (c) represent the 3D of hydrogen bond field diagram that MMP-1 and MMP-9 are, respectively, represented by ribbons; amino acids of abalone peptide involved in hydrogen bonds are represented by thin sticks. Parts (d) represent the 3D of hydrogen bond field diagram that MMP-1 and MMP-9 are, respectively, represented by ribbons; amino acids of abalone peptide involved in hydrogen bonds are represented by thin sticks. Parts (e) and (f) represent that abalone peptide is colored by green and important amino acids of MMP-1 and MMP-9 are colored by black, respectively. The dotted potion represents the hydrogen bonds between them and abalone peptide. Two-dimensional (2D) diagrams of predicted interactions between abalone peptide with amino acid residues of MMP-1 (g) and MMP-9 (h), only displayed interacting residues.

The 3D structure of MMP-1 and MMP-9, and the bioactive effects of abalone peptide were concatenated with Accelrys Discovery Studio 2.1 software and energy minimized with the CHARMm program using steepest descent and conjugate gradient techniques. Water molecules in the protein-crystal structure must be removed before the docking procedure. The CDOCKER results about MMP-1-ATPGDEG and MMP-9-ATPGDEG combination, respectively, were showed in best interaction poses with −56.7684 and −78.7558 kcal/mol of CDOCKER interaction energy (Fig. 7a–d). Abalone peptide has numerous interactions with amino acid residue of MMP-1; further analysis of result showed that there were five generated hydrogen bonds, with the bond length of 2.36, 2.32, 2.05, 2.65, and 2.41 Å with Thr241, Thr241, Tyr237, Tyr237, and Asn180, respectively (Fig. 7e). And four hydrogen bonds were observed between abalone peptide and the Thr109, Asp206, Asp206, and Glu208 residues with the length of 2.18, 2.10, 2.39, and 1.95 Å, respectively (Fig. 7f).

## Discussion

This study demonstrated that abalone peptide is an antiphotoaging agent to attenuate oxidation-related damage induced by UVB to cells via mechanisms involving MAPKs and NF-κB pathways.

The cytotoxic effect of abalone peptide and UVB irradiation were not observed in the HaCaT cells. The over-existence of free radical in the cytoplasmic matrix is an important intrinsic factor for oxidative damage. When human skin is continuously exposed to UV, the expression level of ROS is increased, which is the same as the previous result of Leirós (5) that ROS can fortify the expression of MMP-1 in cells. Moreover, ROS generated by UVB irradiation may induce the expression of MMPs result in photoaging (44). Therefore, research shows that antioxidants such as alga *Corallina pilulifera* methanol (CPM) extract and *aaptamine* from the marine sponge have been extensively blocked ROS generation to use against UVB-induced skin photoaging (45, 46).

ELISA assay demonstrated that UVB mediated aging response of on MMP-1 and MMP-9 upregulation, also caused reduction of type I procollagen in HaCaT cells, those phenomena significantly reversed by abalone peptide in 50 μM of concentration. Many studies on human wound have found that MMP-1 is always expressed by basal keratinocytes that migrate to dermal matrix. Current research suggests that the recovery of type I procollagen is evidence of skin protection due to a significant reduction in type I procollagen biosynthesis in photoaging skin, resulting in loss of skin elasticity (47). This might also be attributed to the key roles of MMP-1 and MMP-9 as the primary phenomenon in UVB-irradiated human skin, and thus we judged UVB irradiation was responsible for type I procollagen destruction in photo-damaged skin, which is consistent with Liu’s result (48).

A research study came up that molecular mechanism of UVB radiation-induced skin damage is complex and involves many signaling molecules that are relevant with stromal collagen, including MMPs, MAPKs, and NF-κB, among others (49). Analyzing from molecular level, oxidative stress generated by excessive ROS may lead to the activation of the MAPKs signaling pathway consisting of ERK, p38 MAPK, and JNK, which further influence the activity of transcription factor AP-1 (Fos/Jun) (20). In human keratinocytes, UVB most likely triggers the activation of JNK via phosphorylation of JNK (50). As shown above, UVB radiation notably promoted JNK phosphorylation in HaCaT cells, serving as an upstream signal to exacerbate additional oxidative damage induced by UVB exposure, which is the same as that UVB irradiation activates ERKs, JNK, and p38 kinases by phosphorylation (29). And abalone peptide treatment partially abolished the UVB-stimulated activation of JNK. Western blot assay analyzed the relative pathway expression of the mechanisms of anti-photoaging and discovered that abalone peptide may significantly decrease the expression of MMP-1 and MMP-9 by suppressing phosphorylation of MAPKs and NF-κB. We observed that NF-κB was upregulated in the UVB group, suggesting that ROS produced by UVB radiation exposure stimulated MMPs expression via NF-κB signaling. In contrast, abalone peptide treatment reduced the effect of NF-κB, same as the result of DNA damage which notably decreased, suggesting that inhibition of this pathway is another mechanism by which abalone peptide exerts antiphotoaging effects.

The main influence factors of the peptide’s biological activity are the sequence and position of amino acid (51). Therefore, we use molecular docking simulation to explore the antiphotoaging structure–activity relationship for peptide with MMP-1 and MMP-9. Most inhibition with less selectivity precisely because active sites of the MMPs are over-conservative (52), while the structures of MMPs are analogical, and the dominating distinction rests in the S1’ pocket. MMP-1 could be inactivated by docking its S1’ and S3’ pockets. Short and narrow is the key feature of the S1’ pocket of MMP-1, including Tyr240, Thr241, His218, Ala183, and Ala234 (53). And the S1’ pocket in MMP-9 for its site specific is referred to as tunnel (54). Abalone peptide generated interaction with Thr241 of S1’ pocket of MMP-1, which means it activated MMP-1 and made inhibitive effort to MMP-1 (Fig. 7g). While abalone peptide was imbed in ‘tunnel’ of MMP-9 (Fig. 7h), we further analyzed the 2D mutual effect of hydrogen bonds of MMP-1 and MMP-9 by contrasting (Tab. 1), and detected that the combine bond parameters of the best pose, both MMP-1 and MMP-9 connected with abalone peptide, are different. It is hydrogen bonds interaction force that plays an important role of stable complex and catalytic reactions in docking. Obviously, MMP-1 with high efficiency exhibited stable interaction by means of four hydrogen bonds (one of them is Thr241 of S1’ pocket of MMP-1), and MMP-9 connected with abalone peptide is tight via aggregation of hydrogen bonds (Glu208: 1; Asp206: 2). This could be explained by abalone peptide’s composition and position of amino acid. Gly of abalone peptide, as the simplest amino acid in them, could provide the space for insertion of enzymatic active site (55). Peptides with Pro and Leu might have high inhibitory MMPs activities in previous study (56), while abalone peptide exactly possess Pro. This might also be attributed to the N-terminal Ala and Pro at the third position of N-terminal which showed stronger hydrophobicity and interaction with MMPs, and C-terminal Gly which showed stronger hydrophilicity and interaction with MMPs.

**Table 1:** Results of ablone peptide docking with MMP-1 and MMP-9, respectively.

Resolution (Å)		MMP-1		MMP-9	
		1.9		1.83	
CDOCKER interaction energy (kcal/mol)		−56.7684		−78.7558	
Types of force	Van der Waals	2		2	
	Attractive charge	1		3	
	Conventional hydrogen bonds	4		4	
	Carbon hydrogen bonds	2		4	
	Unfavorable doner–doner	1		0	
	Unfavorable acceptor-acceptor	1		0	
	Pi-cation	1		0	
	Pi-doner hydrogen bonds	3		0	
	Unfavorable negative-negative	0		2	
Hydrogen bonds		Interacting atoms	Distance (Å)	Interacting atoms	Distance (Å)
		Thr241	2.36	Thr109	2.18
		Thr241	2.32	Asp206	2.10
		Tyr237	2.05	Asp206	2.39
		Tyr237	2.65	Glu208	1.95
		Asn180	2.41		

## Conclusion

This study confirmed that abalone peptide from BABs contains antiphotoaging effects against oxidative stress and protects type I pro collagen. Upon adding this peptide, UVB-induced ROS-free radicals in HaCaT cells were scavenged, which, in turn, attenuated the production in MMP-1 and MMP-9. Thus, abalone peptide may play an anti-photoaging role to protect type I pro collagen and DNA damage via MAPKs and NF-κB signaling. Molecular docking results indicated that abalone peptide’s N-terminal Ala, C-terminal Gly, and Pro at the third position of N-terminal made an important effort to docking active site of MMP-1 and MMP-9, consequently inhibiting their activities. Therefore, abalone peptide is possibly of medicinal application in the prevention and treatment of photoaging, and has the ability to reduce the oxidative stress irradiated by UVB in HaCaT cells system. Hence, it is highly valuable further in the utilization and application of food, cosmetics, and in pharmaceuticals industry.
